# P223: The impact of reminder program on catheter-related blood stream infection rates in an intensive care unit in single center of korea

**DOI:** 10.1186/2047-2994-2-S1-P223

**Published:** 2013-06-20

**Authors:** K-H Park, OJ Choi, HL Lee, ES Shin, GS Kim, IK Lee, SI Jung, BH Cho, HB Kim

**Affiliations:** 1 Office for Infection Control, Chonnam National University Hospital, Gwang-ju, Korea, Republic Of; 2 Medical Intensive Care Unit, Chonnam National University Hospital, Gwang-ju, Korea, Republic Of; 3 Departments of Infectious Diseases, Chonnam National University Hospital, Gwang-ju, Korea, Republic Of; 4 Department of Nursing Graduate School, Chonnam National University Hospital, Gwang-ju, Korea, Republic Of; 5 Departments of Infectious Diseases, 2Seoul National University Hosptia, Seoul, Korea, Republic Of

## Introduction

catheter-related blood stream infection rates (CR-BSIs) are a substantial problem in the intensive care unit.

## Objectives

to evaluate the impacts of reminder program of Medical staffs on the adherence to maximal barrier precautions (MBP), the duration of catheterization (days), and catheter-related blood stream infection rates (CR-BSIs).

## Methods

A simulated control group pretest-posttest design was used for this study. Data were collected from the medical intensive care unit of the C university hospital in Gwangju, during two periods; Period 1 (from January, 2012 to April, 2012) served as the control group who did not received the reminder program, Period 2 (from June, 2012 to September, 2012) served as the experimental group who received the reminder program. The reminder program consisted of MBP adherence exhibit, short message service (adherence feedback to MBP, optimal recommendation, and hand washing), self-report checklist on central venous catheter management and questionnaire to the physicians about the utility of the central venous catheters (CVC) in electronic medical records.

## Results

The participants were 35 physicians who inserted central catheters, 17 nurses and 165 patients (control group 75, experimental group 90) with central catheter insertions for more than 48 hours. The adherence level of MBP was significally increased from 87.7% to 97.9% (p = .026) after implementation of the reminder program. The duration of cathetherization was significally decreased in Period 2 compared Period 1 (from 10.6 to 7.4 days, p = .024). The density incidence of CR-BSI rate was significally decreased in Period 2 compared Period 1 (3.7 to 1.5/1,000 catheter-days, Relative risk 2.51; 95% confidence interval 2.33~2.69, p < .001).

## Conclusion

Reminder Program on Catheter-Related Blood Stream Infection Rates for patients with CVC in an Intensive Care Unit was effective in improving the adherence level of MBP and decreasing the duration of catheterization and CR-BSI rate.

## Disclosure of interest

None declared

